# Green extraction of *Milletia pinnata* oil for the development, and characterization of pectin crosslinked carboxymethyl cellulose/guar gum herbal nano hydrogel

**DOI:** 10.3389/fchem.2023.1260165

**Published:** 2023-09-14

**Authors:** Tipare Bhagyashree Devidas, Sandip Patil, Minaxi Sharma, Nemat Ali, Mohammad Khalid Parvez, Mohammed S. Al-Dosari, Sixi Liu, Baskaran Stephen Inbaraj, Aarti Bains, Feiqiu Wen

**Affiliations:** ^1^ Department of Microbiology, Lovely Professional University, Phagawara, Punjab, India; ^2^ Deparment of Haematology and Oncology, Shenzhen Children’s Hospital, Shenzhen, China; ^3^ Shenzhen Institute of Paediatrics, Shenzhen Children’s Hospital, Shenzhen, China; ^4^ Haute Ecole Provinciale de Hainaut–Condorcet, Ath, Belgium; ^5^ Department of Pharmacology and Toxicology, College of Pharmacy, King Saud University, Riyadh, Saudi Arabia; ^6^ Department of Pharmacognosy, College of Pharmacy, King Saud University, Riyadh, Saudi Arabia; ^7^ Department of Food Science, Fu Jen Catholic University, New Taipei City, Taiwan

**Keywords:** green hydrodistillation, nanohydrogel, antimicrobial, carboxymethyl cellulose, pectin

## Abstract

*Milletia pinnata* oil and *Nardostachys jatamansi* are rich sources of bioactive compounds and have been utilized to formulate various herbal formulations, however, due to certain environmental conditions, pure extract form is prone to degradation. Therefore, in this, study, a green hydrodistillation technology was used to extract *M. pinnata oil* and *N. jatamansi* root for the further application in development of pectin crosslinked carboxymethyl cellulose/guar-gum nano hydrogel. Both oil and extract revealed the presence of spirojatamol and hexadecanoic acid methyl ester. Varied concentrations (w/w) of cross-linker and gelling agent were used to formulate oil emulsion extract gel (OEEG1, OEG1, OEEG2, OEG2, OEEG3, OEG3, OEEG4, OEG4, OEEG5, OEG5), in which OEEG2 and OEG2 were found to be stable. The hydrogel displayed an average droplet size of 186.7 nm and a zeta potential of −20.5 mV. Endo and exothermic peaks and the key functional groups including hydroxyl, amide II, and amide III groups confirmed thermal stability and molecular structure. The smooth surface confirmed structural uniformity. Bactericidal activity against both Gram-positive (25.41 ± 0.09 mm) and Gram-negative (27.25 ± 0.01 mm) bacteria and anti-inflammatory activity (49.25%–83.47%) makes nanohydrogel a potential option for treating various infections caused by pathogenic microorganisms. In conclusion, the use of green hydrodistillation technology can be used to extract the bioactive compounds that can be used in formulation of biocompatible and hydrophobic nanohydrogels. Their ability to absorb target-specific drugs makes them a potential option for treating various infections caused by pathogenic microorganisms.

## 1 Introduction

Nano hydrogels are nanoscale three-dimensional porous structures that are highly stable and soluble and have an affinity for oil and lipophilic compounds. These are composed of cross-linked polymeric networks that enable them to have a large surface area, swell up without dispersing, and have high water-holding capacity (Kaur et al., 2021; Zhan et al., 2021). Nano hydrogels have wide application in drug delivery systems due to their stability, permeability, and better penetration efficiency inside the skin therefore, most widely used in pharmaceutical industries and have wide environmental applications (Ma et al., 2022). The nano hydrogels can be synthesized through diverse methodologies, however, the most stable, economical, and energy-efficient for developing nano hydrogels is a low-energy method that acquires basic agitation such as chemical energy, and yields a small droplet product (Li et al., 2021). The low energy-assisted method entails a two-step process for nano hydrogel formulation. Step one involves formulating an emulsion using key components such as oil, extract, emulsifier, and water, while step two involves the subsequent addition of a crosslinker and gelling agent. The nano hydrogels formulated using natural polysaccharides are biocompatible, biodegradable, and non-toxic and have solubility in water due to their hydrophilic functional groups (Sabzevar et al., 2021).

Furthermore, several herbal extracts due to their remarkable biological properties are also utilized along with naturally occurring polymers for the synthesis of nano hydrogels (Do et al., 2022; Xavier-Santos et al., 2022). The natural polymers act as emulsifiers, binders, and stabilizers and provide a gel-like appearance to the nano hydrogels, however, the herbal extract and oil rich in bioactive compounds are responsible for several biological activities that include antimicrobial, antioxidant, antiseptic, and anti-inflammatory, anti-cancerous properties (Yusuf et al., 2012; Khan et al., 2019; Khan et al., 2021).

In the present study, nano hydrogels are synthesized using *N. jatamansi* root extract and *Milletia pinnata* seed oil in water emulsion. It has been reported that *M. pinnata* seed oil and *N*. *jatamansi* root extract consist of essential bioactive compounds that include karanjin, pongamol, furanoflavonoid, furanoflavones, furanoflavonols, chromenoflavones, flavones, furanodiketones, terpenoids, phenols, sesquiterpenoids, terpenic coumarins, flavonoids, alkaloids, lignans, and neo-lignan, flavones, furanodiketones, terpenoids, phenols, sesquiterpenoids, terpenic coumarins, flavonoids, alkaloids, lignans, and neo-lignan, jatamansone, nardostachone, dihydrojatamansin, jatamansinone, jatamansinol, oroseolol, seselin, valeranal, nardostachin, nardosinone, spirojatamol, jatamols A and B, jatamansic acid, seychelane, seychellene, and calarenol), an alkaloid (actinidine and glaziovine), coumarins (jatamansin or xanthogalin) (Sundrarajan et al., 2015; Saroya et al., 2018; Bose et al., 2019). All these compounds have remarkable antimicrobial, anti-inflammatory, anti-dermatophytic, anti-hyperglycemic, anti-lipid peroxidase, antispasmodic, antiviral, antioxidant effects, and analgesic properties. Various approaches have been explored for the extraction of bioactive compounds, however, certain extraction techniques are found to be hazardous for the environment and human health. Therefore, the demand of green and environmental friendly techniques are increasing for the extraction of vital bioalogical active compounds (Chemat et al., 2019; Yusuf and Shahid, 2022). Hydrodistillation is one of the widely utilized green technology to extract bioactive compounds and the oils from plant derived materials (Awad et al., 2021). In this technique organic solvents are completely eradicated and it could be performed prior to dehydration of the plant derived materials. Primarly hydrodistillation process is composed of three important physicochemical steps including hydrodiffusion, hydrolysis and decomposition by heating process (Chemat et al., 2019; Awad et al., 2021; Yusuf and Khan, 2022). In addition, over the various other extraction techniques, hydrodistillation approach revealed remarkable advantanges in therms of bioactivity of biological active compounds. Therefore, in this research hydrodistillation process was used to extract oil from the seed sample to ensure maximum biological activity (Fagbemi et al., 2021).

Furthermore, *M*. *pinnata* seed oil and *N*. *jatamansi* root extract are used to cure various skin infections like eczema, vitiligo, and psoriasis, and are used in cosmetics, as well as can be applied to burns and skin inflammation (Abbasi and Tahir, 2020; Singh, 2020; Mitra et al., 2021; Wang et al., 2021). The natural polymer pectin and guar gum are used as emulsifiers, binders, and stabilizers that provide the formulation with its gel-like appearance in the present study (Kedir et al., 2022; Lopez-Martinez et al., 2022; Sayed et al., 2022). However, limited research is available regarding the evaluation of synergistic bioactivity of the combination of both *M. pinnata oil* and *N. jatamansi* root extract for nano hydrogel formulation.

Therefore, in the present study, *M*. *pinnata* oil and ethanol extract of *N*. *jatamansi* root were employed to develop nano hydrogels exhibiting antimicrobial, antibiofilm, and anti-inflammatory properties. Additionally, we have provided data of various analysis technique to characterize the oil and extract, respectively. To provide insight of the formulated nano hydrogel was also characterized using Fourier transform infrared spectroscopy (FTIR), scanning electron microscope (SEM), thermogravimetric analysis (TGA), differential scanning calorimetry (DSC), droplet size, and zeta potential. Overall, this study represents a significant contribution towards the advancement of techniques that hold potential benefits for human health as well as extensive pharmacological and food industry applications.

## 2 Materials and methods

### 2.1 Materials

Seeds of *M*. *pinnatta* were obtained from Amritash Herbocare, Ellenabad, Haryana, India, and *N*. *jatamansi* roots were procured from Namo Organics, Punjab, India. Lecithin, carboxymethyl cellulose, pectin, guar gum, Mueller Hinton Agar, Mueller Hinton Broth, dimethyl sulphoxide (DMSO), tween 80, streptomycin, sodium chloride, ethanol, acetic acid, crystal violet dye, n-Hexane were obtained from Hi-Media India Pvt. Ltd., Maharashtra, India. The pathogenic microbial test strains (bacterial strains), i.e., *Escherichia coli* (MTCC 443), and *Staphylococcus aureus* (MTCC 96), were collected from IMTECH (Institute of Microbial Technology) Chandigarh, India. The master cultures were revived in nutrient broth and kept at 4°C for further use.

### 2.2 Methods

#### 2.2.1 Green extraction of *M*. *pinnata* oil

The extraction of oil from *M*. *pinnata* seed was carried out by the hydro distillation process described by Elyemni et al. (2019). Briefly, 20 g of seed powder was taken in a round bottom flask and dispersed in 80 mL ethanol. The reaction mixture was then kept for 7 h at 70°C in the Clevenger apparatus (BORO G Borosilicate 3.3 glass). The Clevenger apparatus consists of a boiling flask, a condenser, and a funnel for the separation of the aqueous phase from the oil phase. The oil phase was separated by drying over anhydrous sodium sulfate in a rotary evaporator (IKA^®^ RV 10 rotary evaporator, New Delhi, India). The oil was then stored in a dark bottle at room temperature for further use.

#### 2.2.2 Preparation of *N*. *jatamansi* root extract

The extract preparation of *N*. *jatamansi* root was carried out by the modified solvent extraction method followed by Chawla et al. (2019). Herein, the 50 g dried *N*. *jatamansi* roots were crushed to a fine powder utilizing a mechanical grinder (Vevor, 2500 g electric grain miller grinder, high-speed 3750W). The fine powder was dissolved in 500 mL of absolute ethanol and kept in an orbital shaker for 72 h at 30°C. The sample was then filtered utilizing Whatman no. 1 filter paper and the supernatant was kept for evaporation at 4 °C in the refrigerator for 2 weeks. The extract was then collected in glass vials for further analysis.

#### 2.2.3 Identification of seed oil and root extract bioactive compounds

The identification of essential fatty acids and bioactive compounds present in seed oil and root extract was carried out by Gas chromatography-mass spectrometry (Thermo Fisher Scientific, Waltham, MA, United States). The instrument was set with GC 1300 gas chromatography, autosampler (TriPlus RSH), TSQ Duo Mass selective quadrupole detector, and Trace Gold TG-5MS column (40 m length, internal diameter 0.15 mm, and film thickness 0.15 m). The analysis of compounds present was done by diluting each sample in n-hexane at a ratio of 1:99. The initial temperature was kept at 60°C for 60 s to 180°C for 3 min and the final temperature was kept at 240°C for 12 min. The ramp rate was maintained constant at a temperature of 10°C/min and the complete analysis was kept for 34 min. The program was run with the linear velocity of the helium carrier gas at the rate of 0.7 mL/min. The electron impact was kept at 70 eV and used as ion source temperature with 250°C of transfer line temperature at 230°C. The sector of the mass analyzer was set to scan from m/z 45 and 450. Gas chromatographic and mass spectrometric data were processed by using Xcalliber software.

#### 2.2.4 Preparation of nano hydrogel

Formulation of nano hydrogel from oil-in-water emulsion was carried out by the low-energy-assisted method described by (Thakur et al., 2023). Briefly, 1% each carboxymethyl cellulose, lecithin, tween 80 and 100 mg *N. jatamansi* root extract were added in a 100 mL volume of distilled water and stirred simultaneously using a magnetic stirrer at 1800 rpm for 1 h 30 min at room temperature. Furthermore, formulation of emulsion-based guar gum nano hydrogel was done by covalent bonding using pectin as a cross-linker. Herein, nano hydrogel pectin and guar gum at different concentrations (pectin: guar gum, w/w) 1:1 (OEEG1), 1:1.5 (OEEG2), 1.5:1 (OEEG3), 2:1 (OEEG4), 1:2 (OEEG5) was added and stirred further at 800 rpm for 30 min. Nano hydrogel without the addition of extract was also prepared with the same pectin and guar gum concentration (pectin: guar gum) 1:1 (OEG1), 1:1.5 (OEG2), 1.5:1 (OEG3), 2:1 (OEG4), 1:2 (OEG5). The samples were stored in glass vials at temperatures ranging from 4°C to 7°C for further analysis.

#### 2.2.5 Characterization of nano hydrogel

##### 2.2.5.1 Droplet size and electrokinetic potential

The size distribution of particles and electrokinetic potential of nano hydrogel were carried out by the method followed by Moradi et al. (2019) using a Zetasizer Nano Scale (Zetasizer Nano ZS, Malvern Instruments Ltd. Malvern, WR14 1XZ, UK). Briefly, a nano hydrogel sample (0.1 mL) was dissolved in phosphate buffer (0.05M, pH 7.0) before analysis at a temperature of 25 °C followed by an ultrasonication process and finally subjected to average droplet size and zeta potential measurement.

##### 2.2.5.2 Scanning electron microscopy (SEM)

The nano hydrogel surface structure was examined by using FESEM (field emission scanning electron microscopy) (JEOL-JSM-7610F, Akishima, Tokyo, Japan). Briefly, a 6 mg freeze-dried nano hydrogel sample was fixed on a carbon-coated copper grid on a glass slide and to improve the conductivity coating of gold was done by a sputtering method for 120 s at 30 mA. Samples of photomicrographs were noted and a high-quality image was obtained at 2000 × with 10 kV accelerating voltage.

##### 2.2.5.3 Differential scanning calorimetry (DSC)

The physiochemical interaction of compounds present within the nano hydrogel was evaluated by the method followed by Shahbazizadeh et al. (2022) utilizing a differential scanning calorimeter equipped with thermocouple-based temperature sensors and a nickel-chromium sample plate (Perkin Elmer, Massachusetts, United States). Briefly to obtain the measurement the nano hydrogel sample was heated in a calorimeter in the presence of 99.9% N_2_ (inert atmosphere) at a temperature ranging from 30 C to 450°C with 10°C/min rate of heating.

##### 2.2.5.4 Fourier-transform infrared spectroscopy (FTIR)

FTIR (Perkin Elmer, Spectrum 2, Ueberlingen, Germany) was carried out to confirm the functional group present in the nano hydrogel following the method as described by Arora et al. (2021). Briefly, a 10 mg nano hydrogel sample was mixed with standard potassium bromide, and the spectra thus obtained using air as background in the range of 4,000–400 cm^−1^ mid-infra region was studied using Spectrum 10 Software.

#### 2.2.6 Antimicrobial activity

The antimicrobial susceptibility of nano hydrogel against pathogenic gram-positive and gram-negative bacteria was carried out by agar well diffusion method as described by Sepahvand et al. (2021). Briefly, MHA (Mueller Hinton agar) plates were prepared and wells were made using sterile cork borer. The test microorganisms both gram-positive *S. aureus* and *E. coli* were spread over the plates using a sterile cotton swab and oil, extract, formulated nano hydrogel, antibiotic (positive control), and 5% DMSO (negative control) were added in each well. The plates were then incubated at 37°C for 24 h and results were obtained and zone of inhibition (ZOI) mm. Furthermore, minimum inhibitory concentartaion and minimum bactericidal concentartion of oil, extract and nanohydrogel was done by broth microdilution assay (Bains et al., 2023). Herein, different cocentration of each sample taken in microtitre plate and MHB was taken as test medium for pathogenic bacteria and kept for incubation at 37°C for 24 h. The MBC of each microorgansim was observed by subculturing the MHA plates.

#### 2.2.7 Biofilm activity

The antibiofilm activity of formulated nano hydrogel was carried out by the ELISA plate method outlined by (Bouaouina et al., 2022) with slight modifications. Briefly, the test cultures *E. coli,* and *S. aureus* were revived in Mueller Hinton broth, and incubation was done for 12–18 h. The culture (50 µL) was added in the first well followed by the addition of nano hydrogel (100 µL) and then dilution was made up to the 11th well while the 12th well was taken as control (no addition of nano hydrogel). To each well 50 µL of culture was added and the microtiter plates were incubated at 37 C for 72 h. The culture was then removed from each well and wells were rinsed with 0.9% NaCl and dried for 15 min at 60°C followed by the addition of crystal violet and incubation at 27 C for 15 min. The retained crystal violet by cell was obtained by rinsing the wells with 0.9% NaCl and 30% acetic acid. Absorbance was observed at 595 nm using an ELISA plate reader. The percentage inhibition was calculated by following Equation 1.

$$Inhibition\;\left(\%\right)=\left[\left(\frac{\mathrm{optical density of negative control - optical density of sample}}{\mathrm{optical density of negative control}}\right)\times 100\right]$$ (1)

#### 2.2.8 Anti-inflammatory activity

The anti-inflammatory activity of formulated nano hydrogel was evaluated by the albumin denaturation method followed by (Qamar et al., 2021). Herein, bovine serum albumin (0.5 mL) and 0.85% NaCl (2.5 mL) was added to test tubes containing nano hydrogel of different concentration (50, 100, 150, and 200 mg/mL). The tubes were incubated at room temperature for 20 min followed by incubation at 70 °C for 5 min and then allowed to cool. Phosphate-buffered saline (pH 6.4) was added to each tube and results were observed at 660 nm using a spectrophotometer. The percentage inhibition was calculated by using the following equation:

$$BSA\;Denaturation\;\left(\%\right)=\left[\left(1-\frac{{Sample}_{abs}}{C{ontrol}_{abs}}\right)\times 100\right]$$ (2)

#### 2.2.9 Statistical analysis

All the experiments performed in the present study was carried out in triplicates and the data obtained was represented as a mean of three independent determination ± standard deviation. The obtained data were subjected to a *t*-test and one-way ANOVA to calculate the statistically different mean values at *p* < 0.05. Microsoft^®^ Excel_®_, 2021 (Microsoft Corporation, Redmond, Washington, United States) was used to compare mean values estimated by the critical difference (CD) value.

## 3 Results

### 3.1 Isolation of compounds

**FIGURE 1:**
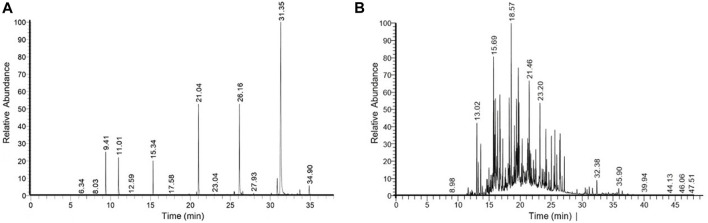
GC-MS chromatogram of bioactive components of **(A)** Milletia pinnata seed oil and **(B)** Nardostachys jatamansi root extract.

The phytocompounds isolated from the *M*. *pinnata* oil and *N*. *jatamansi* extract are shown in Figures 1A, B. The molecular formula, retention time, and chemical profiling of isolated compounds are depicted in Tables 1, 2. The *M*. *pinnata* oil peaks were recorded at the retention times 9.41, 11.01, 15.34, 21.04, 26.16, 30.15, and 31.34 and revealed the presence of hexanoic acid, methyl ester, octanoic acid, 4-methyl-, methyl ester (Capric acid), aniline, nonanoic acid, methyl ester, octanoic acid methyl ester, decanoic acid, methyl ester (capric acid), undecanoic acid, 2-methyl-, undecanoic acid, 2-methyl-, pentanoic acid, methyl ester (palmitic acid), dodecanoic acid, methyl ester (lauric acid), tridecanoic acid, methyl ester, tetradecanoic acid, 12-methyl-, methyl ester, hexadecanoic acid methyl ester, and pentadecanoic acid, methyl ester (palmitic acid). The *N*. *jatamansi* extract peaks were recorded at the retention times 13.02, 13.64, 15.69, 16.74, 17.20, 18.23, 19.27, 19.41, 19.49, 19.71, 19.83, 20.34, 21.46, 21.60, 23.21, 24.14, 25.61, and 26.47 which revealed the presence of cyclopentaneacetaldehyde, 2-formyl-3-methyl-à-methylene, 2-(1-methylcyclopropyl) aniline, muurolene, naphthalene, 1, 2, 3, 5,6, 7, 8, 8 a-octahydro-1,8a-di methyl-7-(1- methylethenyl)-, (1R-(1à,7á,8aà)-, panasinsen, 7-epi-a-eudesmol, à-maalien, (−)-à-panasinsen, .tau.-cadinol, à-cadinol, spirojatamol, isoaromadendrene epoxide, 6-Isopropenyl-4,8a-dimethyl-1,2, 3,5,6,7,8,8a-octahydro-naphthalen -2-ol, Calerene epoxide, patchouli alcohol, 1(2H)-naphthalenone, octahydro-4a,8a-dimethyl-7-(1-m ethylethyl)-, (4aR-(4aà,7á,8aà))-, á-longipinene, geranyl-à-terpinene, (1aR,4S,4aR,7R,7aS,7bS)-1,1,4,7 -tetramethyldecahydro-1H-cyclo propa(e)azulen-4-ol (globulol), tricyclo (5.1.0.0 (2,4))oct-5-ene-5- propanoic acid, 3,3,8,8-tetramethyl, hexadecanoic acid, methyl ester, hexadecanoic acid, 2-methyl-, pentadecanoic acid, methyl ester, hexadecanoic acid, 2-methyl-, methyl ester, hexadecanoic acid, ethyl ester, 11-octadecenoic acid, methyl ester, (Z)-, ethyl oleate. The compounds globulol, capric acid, palmitic acid, and lauric acid isolated from oil and root extract possess antioxidant, antiproliferative, anti-inflammatory, and antimicrobial activity. The present results agreed with the results obtained by Rekha et al. (2021), Sami et al. (2022), Arya et al. (2022) who isolated the different phytocompounds from *M*. *pinnata* seed oil, and *N*. *jatamansi* root extract by using GCMS technique and evaluated for their biological activities.

**TABLE 1:** Identification of Compounds in *Milletia pinnata* oil using Gas Chromatography-Mass Spectrometer.

Compound name	Retention time (min)	Molecular formula
Hexanoic acid, methyl ester	9.41	C_7_H_14_O_2_
Aniline	11.01	C_6_H_7_N
Octanoic acid methyl ester	15.34	C_9_H_18_O_2_
Decanoic acid, methyl ester (Capric acid)	21.04	C_11_H_22_O_2_
Pentanoic acid, methyl ester (palmitic acid)	26.16	C_16_H_32_O_2_
Tetradecanoic acid, 12-methyl-, methyl ester	30.15	C_16_H_32_O_2_
Pentadecanoic acid, methyl ester (palmitic acid)	31.34	C_16_H_32_O_2_

**TABLE 2:** Identification of Compounds in *Nardostachys jatamansi* root extract using Gas Chromatography-Mass Spectrometry.

Compound name	Retention time (min)	Molecular formula
Cyclopentaneacetaldehyde, 2-formyl-3-methyl-à-methylene	13.02	C_10_H_14_O_2_
2-(1-Methylcyclopropyl) aniline	13.64	C_10_H_13_N
Naphthalene, 1,2,3,5,6,7,8,8a-octahydro-1,8a-di methyl-7-(1-methylethenyl)-, [1R-(1à,7á,8aà)]-	16.74	C_15_H_24_
7-epi-a-Eudesmol	17.20	C_15_H_26_O
Spirojatamol	18.23	C15H26O
Isoaromadendrene epoxide	19.27	C_15_H_24_O
geranyl-à-terpinene		
(1aR,4S,4aR,7R,7aS,7bS)-1,1,4,7 -Tetramethyldecahydro-1H-cyclo propa [e]azulen-4-ol (Globulol)	21.46	C_15_H_26_O
Tricyclo [5.1.0.0 (2,4)]oct-5-ene-5- propanoic acid, 3,3,8,8-tetramethyl	21.60	C_15_H_22_O_2_
Hexadecanoic acid, methyl ester	23.21	C_17_H_34_O_2_
Hexadecanoic acid, 2-methyl-, methyl ester	24.14	C_18_H_36_O_2_
11-Octadecenoic acid, methyl ester, (Z)-	25.61	C_19_H_36_O_2_
Ethyl oleate	26.47	C_20_H_38_O_2_

### 3.2 Characterization of nano hydrogel

#### 3.2.1 Physical characterization

The synthesis of the emulsion was carried out by a low-energy assisted method of emulsion polymerization by utilizing root extract, carboxymethyl cellulose as binder, lecithin a negatively charged lipid mixture of phospholipids, and non-ionic surfactant Tween-80. During the low-energy process, the appearance of milky white color confirmed the emulsion formulation. As well, for micellar polymerization within the emulsion moiety gelling and crosslinking agents were added, which resulted in the aggregation of the emulsion droplets in spherical micelles. The process continued until the surface tension declined and the invasion of hydrophobic monomers in the vicinity of the micelle. The binding sites of invaded hydrophobic monomers caused exhaustion in all the monomeric droplets, which helped in the enhancement of the structural micelle. The appearance of the cream-yellow color formulation confirmed the formation of the gel structure. Furthermore, with increasing concentration of the gelling agent the viscosity, cohesiveness, and stickiness were increased, hence among all formulated concentrations, OEEG2 and OEG2 were found to be homogenous and stable, which were then further characterized using various analytical techniques.

#### 3.2.2 Droplet size and electrokinetic potential

**FIGURE 2:**
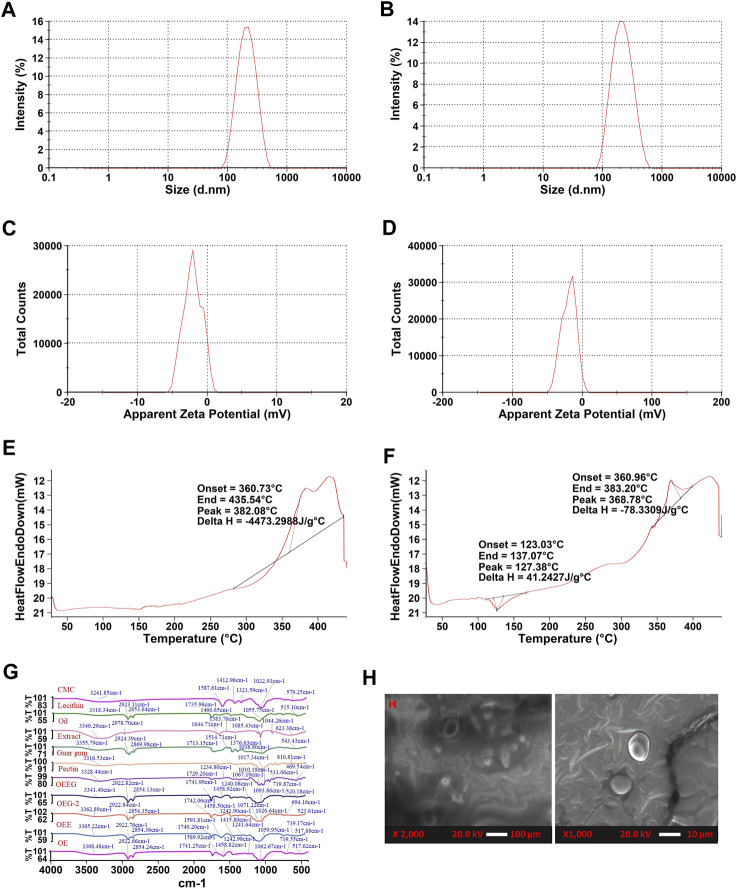
**(A)** The particle size of nanoemulsion-based with extract nano hydrogel (OEEG), **(B)** The particle size of oil emulsion nano hydrogel (OEG-2), **(C)** Zeta potential of nanoemulsion-based with extract nano hydrogel (OEEG), **(D)** Zeta potential of Oil emulsion nano hydrogel (OEG-2), **(E)** Differential scanning calorimetry of nanoemulsion-based with extract nano hydrogel (OEEG), **(F)** Differential scanning calorimetry of oil emulsion nano hydrogel (OEG-2), **(G)** FTIR spectra of Carboxymethyl cellulose (CMC), Lecithin, Milletia pinnata oil (Oil), Nardostachys Jatamansi root extract (Extract), Guar gum, Pectin, Oil in water nanoemulsion with extract nano hydrogel (OEEG), Oil emulsion nano hydrogel-2 (OEG-2), Oil in water nanoemulsion with extract (OEE), and Oil in water nanoemulsion (OE); **(H)** SEM images of formulated nano hydrogel of OEEG and OEG-2.

The hydrogel samples were evaluated for their droplet size and electrokinetic potential and the results are represented in (Figures 2A–D). In the present study, the OEG2 and OEEG2 showed particle sizes of 196.7 nm and 186.7 nm. The size of polymeric nanoparticles ranges from 100 to 1,000 nm providing a stable colloidal dispersion (Jyothi et al., 2017; Saha et al., 2023). The zeta potential was carried out to measure the surface charge of the formulated hydrogel. Herein, control and functional hydrogel exhibited −20.5 mV and −19.3 mV surface charge distribution, respectively. Negative surface charge in the nano hydrogel matrix is mainly attributed to the polyanionic nature of the polysaccharides used to stabilize the nano hydrogel. As well, during gel formulation, polymers added to the emulsion system adsorbed onto the particle surfaces. Appropriate adsorption of the polymers exhibits the thickness and improves the steric repulsion between the polymer layers, which resulted in a negative surface charge of nano hydrogel. Likewise, the higher stability of hydrogels correlates to the zeta potential greater than +25 mV and less than −25 mV. At larger zeta potential, due to stable colloidal dispersion and strong repulsion of the charged droplet within them, there is an overcome in the natural tendency of hydrogel to aggregate (Jyothi et al., 2017). The present results are in accordance with the results of Jyothi et al. (2017), and Saha et al. (2023), who studied the effect of nano hydrogel formulated by utilizing *Crossandra infundibuliformis* extract and polyherbal flowers extract.

#### 3.2.3 DSC (differential scanning calorimetry)

Differential Scanning Calorimetry analysis was performed to comprehend the thermal stability and phase transition behavior of nano hydrogel and the results are represented in (Figures 2E, F). It has been observed that both sample OEG2 represents both endothermic and exothermic peaks at denaturation temperatures 127.38°C, 368.78°C with onset temperatures at 123.03°C, 360.96°C and end set temperature at 137.07°C and 383.20 °C respectively. However, OEEG2 showed an exothermic peak only at a denaturation temperature of 382.08°C, onset temperature at 360.73°C, and end set temperature of 435.54 °C. The endothermic peak in OEG2 is due to the loss of water (Algharib et al., 2020). The exothermic peak can be attributed to the crystalline temperature of the nano hydrogel as well as the decomposition of polysaccharides present in the formulation (Pal et al., 2019). The present results lined with the results of Hajidayor et al. (2023), and Singh et al. (2023) who studied the thermal stability of cellulose-based nano hydrogels from *Elaeis guineensis* and nano hydrogel formulated utilizing *Beta vulgaris* extract.

#### 3.2.4 Fourier-transform infrared spectroscopy (FTIR)

The FTIR spectral peaks of oil in water nano emulsion-based nano hydrogel were recorded at 3341.40, 2922.82, 2854.13, 1714.99, 1598.74, 1458.74, 1240.08, 1093.86, 719.87, and 520.18 cm^−1^ are represented in Figure 2G. The broad peak observed around 3341.4 cm^−1^ represents the hydroxyl group present in the extract, oil, pectin, lecithin, and guar gum. In nano hydrogel, the OH group represents the water molecules which is directly related to the hydrophilic nature of the hydrogel (Altaf et al., 2021). The peak observed at 1598.74 cm^−1^ occurred in the nano hydrogel due to carboxy methyl cellulose indicating the presence of the Amide II group, and the peak at 1240.08 cm^−1^ is due to the pectin and extract that corresponds to the presence of Amide III bend (Slayutsky and Bertuzzi, 2019). The peak observed at 520.18 cm^−1^, 2922.82 cm^−1^, 2854.13 cm^−1^, and 1458.92 cm^−1^ is due to halogen compounds and C-H stretching. The peaks 1353.17 cm^−1^, 1353.17 cm^−^1, 1071.22 cm^−1^, and 813.58 cm^−1,^ 523.61 cm^−1^ in the OEEG2 showed a decreased ratio of a peak on the addition of extract that may be due to increasing in cross-linkage (Mansur et al., 2008). The present results are in line with the results obtained by Lin et al. (2021), who observed similar results in herbal-loaded nano hydrogel samples.

#### 3.2.5 Scanning electron microscope (SEM)

The characteristics of formulated nano hydrogel were observed at 2000x as shown in Figure 2H **.** The nano hydrogel sample OEG2 showed a smooth surface devoid of surface features while sample OEEG2 showed a smooth structure with a uniform dispersion of extract and increased cross-linking density. The smooth surface is due to the −OH group and the increased cross-linking density is due to the emulsifier (guar gum) and extracts (Rahmatpour and Soleimani, 2021).

### 3.3 Antimicrobial activity

**FIGURE 3:**
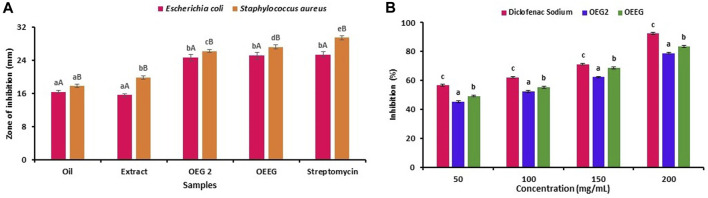
The results were expressed as mean ± standard deviation of >3 independent replicates and error bars represent the standard deviation from mean values, while different lowercase (a–e) and uppercase letters **(A** and **B)** above each bar represent significantly different (*p* < 0.05) from each other.

The antimicrobial susceptibility, MIC and MBC of oil, extract, nano hydrogel sample OEG2 (control) and OEEG2 was studied against gram-positive and gram-negative bacteria and results are represented in Figure 3A; Table 3. Herein, all the samples exhibited a significant (*p* < 0.05) higher zone of inhibition against Gram-positive bacteria *S. aureus* (17.78 ± 0.42–29.47 ± 0.53) as compared to Gram-negative bacteria *E. coli* (16.34 ± 0.32–25.41 ± 0.67). In addition, against *E. coli* extract and oil showed significant (*p* < 0.05) difference in terms of zone of inhibition, while, OEG2 and OEEG2 showed comparable to positive control. Furthermore, MIC and MBC values for *S. aureus* ranges from 25 to 0.78 μL/mL and 12.5–0.39 μL/mL and for *E. coli* the values ranges from 25 to 1.56 μL/mL and 12.5–0.78 μL/mL respectively. The susceptibility of nano hydrogel against test microorganisms was due to the action of nano gels against cell membranes resulting in its damage. The cellular components come in contact with the surface of the gel and this leads to the ultimate death of the cell (Hamidi et al., 2023). The porous structure of nano hydrogel is another contributing factor that facilitates the release of essential phytocompounds present in extract and oil. These components when penetrating the cell membrane structure specifically target the ribosomes and result in the inhibition of protein synthesis (Graf and Wilson, 2019). The obtained results are consistent with the findings reported by Talodthaisong et al. (2020) who formulated guar gum hydrogels crosslinked with borax and loaded with silver nanoparticles to evaluate their antimicrobial properties against *E. coli* and *S. aureus*.

**TABLE 3:** Total MIC and MBC of oil, extract, and formulated nanohydrogel.

Microorganisms	MIC (µL/mL)			
	Oil	Extract	OEG2	OEEG
*Escherichia coli*	25	12.5	6.25	1.56
*Staphylococcus aureus*	25	12.5	3.12	0.78

Microorganisms	MBC (µL/mL)			
	Oil	Extract	OEG2	OEEG
*Escherichia coli*	12.5	6.25	3.12	0.78
*Staphylococcus aureus*	12.5	6.25	1.56	0.39

### 3.4 Antibiofilm activity

The antibiofilm activity of nano hydrogel is shown in Table 4. The nano hydrogel showed a remarkable percentage of biofilm inhibition against Gram-positive bacteria *S. aureus* (76.78%) and Gram-negative bacteria *E. coli* (72.47%). The wide range of antibiofilm activity of nano hydrogel is due to the interaction of nano hydrogel with the negatively charged membrane of microorganisms resulting in the distraction of the membrane’s osmotic balance leading to cell lysis. The nano hydrogels interact electrostatically as well hydrophobically with several components including lipoproteins, lipopolysaccharides, and phospholipids. This interaction results in the adsorption and immobilization of the bacteria on the surface of nano hydrogels. The nano hydrogel may disrupt the process of protein synthesis by binding with bacterial ribosomes thereby interfering with protein transcription and resulting in the inhibition of bacterial activity (Khan et al., 2021; Qiu et al., 2021).

**TABLE 4:** Assessment of antibiofilm activity of a formulated nano hydrogel against food pathogens^a^.

Microorganism	Antibiofilm activity (%)
*Escherichia coli*	72.47 ± 0.98^a^
*Staphylococcus aureus*	76.78 ± 0.73^b^

^a^ Data are presented as mean ± SD (*n* = 3).

Mean values within a column with different lowercase superscripts (a-b) are significantly different (*p* < 0.05) from each other based on Duncan’s multiples rage test.

### 3.5 Anti-inflammatory activity

The antiphlogistic properties of formulated nano hydrogel are represented in Figure 3B. Briefly, the four different concentrations (50, 100, 150, and 200 mg/mL) of the sample were tested and it has been observed that OEEG2 showed a significant difference (*p* < 0.05) in comparison to control OEG2 and positive control diclofenac sodium for each concentration. The OEEG2 showed percentage inhibition ranges from 49.25% to 83.47% while OEG2 and diclofenac sodium showed percentage inhibition ranges from 45.43% to 78.92% and 56.57%–92.48% respectively. The nano hydrogel OEEG2 showed less inhibition in comparison to positive control diclofenac sodium due to the interaction of phenolic compounds including methyl ester, and octadecanoic acid that causes a conformational change in the structure of proteins resulting in a decrease in surface hydrophobicity (Ferreira et al., 2020). Another reason for anti-inflammatory activity might be the presence of guar-gum which control the release of several anti-inflammatory agents, pectin that inhibits phagocytosis, and chemotaxis, which affect the release of anti and pro-inflammatory cytokines by macrophages, and immune modulatory activity by improving macrophage function (Thombare et al., 2016; Kedir et al., 2022).

## 4 Conclusion

In conclusion, this study successfully isolated bioactive compounds from *M. pinnata* oil and *N. Jatamansi* root extract. GC-MS analysis identified various compounds with potential biological activities, including antimicrobials, and anti-inflammatory agents. A nano hydrogel incorporating these compounds was synthesized using a low-energy-assisted emulsion polymerization method. The nano hydrogel exhibited homogeneity, stability, and a particle size of 196.7 nm, with a strong anionic nature suitable for pH-sensitive applications. Characterization techniques confirmed the thermal stability and phase transition behavior of the nano hydrogel, as well as the presence of functional groups and interactions between components. Microscopic analysis revealed a smooth surface and increased cross-linking density. The nano hydrogel demonstrated promising antimicrobial activity against both Gram-positive and Gram-negative bacteria, effectively inhibiting biofilm formation. It also exhibited anti-inflammatory properties by inhibiting protein denaturation. These findings highlight the potential of the isolated compounds and the developed nano hydrogel for various applications in medicine and biotechnology. Further research is needed to explore their therapeutic potential and optimize their utilization. Overall, this study contributes to the growing field of natural compound isolation and nanomaterial development, providing valuable insights for future advancements in healthcare and biotechnological applications.

## Data Availability

The original contributions presented in the study are included in the article/supplementary material, further inquiries can be directed to the corresponding authors.
